# Effect of Extended Lipid Core on the Hemodynamic Parameters: A Fluid-Structure Interaction Approach

**DOI:** 10.1155/2022/2047549

**Published:** 2022-03-17

**Authors:** Morteza Teymoori, Mahmood Reza Sadeghi, Mohsen Rabbani, Mehdi Jahangiri

**Affiliations:** ^1^Department of Biomedical Engineering, Faculty of Engineering, University of Isfahan, 81746-73441 Isfahan, Iran; ^2^Department of Mechanical Engineering, Shahrekord Branch, Islamic Azad University, Shahrekord, Iran

## Abstract

Myocardial infarction is one of the leading causes of death in the developed countries. A majority of myocardial infarctions are caused by the rupture of coronary artery plaques. In order to achieve a better understanding of the effect of the extension of the lipid core into the artery wall on the change of flow field and its effect on plaque vulnerability, we have studied the hemodynamic parameters by utilizing a finite element method and taking into account the fluid-structure interaction (FSI). Four groups of stenosis models with different sizes of lipid core were used in the study. The fully developed pulsatile velocity profile of the right coronary artery was used as the inlet boundary condition, and the pressure pulse was applied as the outlet boundary condition. The non-Newtonian Carreau model was used to simulate the non-Newtonian behavior of blood. Results indicate that the extension of the lipid core into the artery wall influences the flow field; subsequently, creates favorable conditions for additional development of the lipid core which can lead to a higher risk of plaque rupture.

## 1. Introduction

Cardiovascular diseases such as atherosclerosis are the major cause of death in the developed countries [1]. Atherosclerosis is an inflammatory disease of large and medium-sized arteries which is initiated by endothelial cell (EC) dysfunction and increased infiltration of the atherogenic particles such as low density lipoproteins (LDLs) and monocytes into the intimal layer [2]. Possible causes of EC dysfunction include high blood cholesterol, smoking, hypertension, and diabetes. When LDL particles enter the intima, they become oxidized [3]. Monocytes engulf oxidized LDLs (oxLDLs) and form inflammatory macrophage cells. By further LDL aggregation, macrophage formation and some stimulated signaling pathways, the inflamed lesion starts to grow and form lipid rich plaque [4]. The formed plaque may lead to the narrowing of the artery lumen (stenosis) and alteration of local flow dynamics [5]. Disturbed flow dynamics in the stenosed artery has been shown to be associated with further plaque growth and increased plaque vulnerability to erosion or rupture [6, 7]. However, rupture-prone plaques are not necessarily associated with severe luminal narrowing. In such plaques, the inflammation and enzymatic activity lead to the extension of the plaque into the underlying arterial wall layers. So called complex plaques are characterized by thin fibrous caps, large lipid core with inflammatory cells, and the extension of this lipid core to tunica media and rupture of the internal elastic lamina [8]. The increased amount of inflammatory cell leads to higher matrix metalloproteinases (MMPs) production. MMPs are associated with extracellular matrix (ECM) degradation and increased plaque vulnerability [9].

It has been accepted that in addition to biological factors, the hemodynamic parameters in the arterial lumen are also very effective factors in plaque growth and its vulnerability. It was first reported by Caro [10], Friedman et al. [11], and Ku et al. [12] that atherosclerotic plaques are more common in the areas with low and oscillatory shear stress. The effects of the wall shear stress (WSS) have been further studied by a number of researchers [10, 13–17]. According to these studies, low and oscillatory WSS can affect the gene expression and phenotype of the ECs and induce higher migration of the atherogenic particles to the inflamed lesion. From a hemodynamics point of view, this is further augmented by the increased concentration of atherogenic particles in the areas of low and oscillatory WSS (i.e. areas with high OSI (oscillatory shear index) values) [10, 18, 19]. On the other hand, WSS with a level above 40 Pa is able to damage ECs [20], and a WSS of 100 Pa is able to strip ECs and enhance the process of thrombogenesis [21]. Since the role of the lipid core extension into the arterial wall in changing the flow field and its effects on the vulnerability of the plaque has not been explored in previous studies, in the current study, we aim to investigate these effects under physiological condition of vascular blood flow. To do so, we simulated pulsatile blood flow through a stenotic coronary artery and its interaction with a vulnerable plaque. Numerous studies have investigated the flow dynamics in arteries and have shown the importance of a non-Newtonian blood model in the model fidelity [22–27]. Thus, to consider the non-Newtonian behavior of blood, Carreau model was used in the present study. The variation of hemodynamic parameters around the plaque were studied in respect to the stenosis severity and the volume of the lipid core in 4 main groups.

## 2. Materials and Methods

### 2.1. Governing Equations

The flow was assumed to be incompressible, laminar, non-Newtonian, and pulsatile. As governing equations, Continuity and the Navier-Stokes equations with arbitrary Lagrangian-Eulerian (ALE) formulation were used as follows:

(1) $$\begin{array}{c} \nabla ∙V_{f}=0,\\ \rho_{f}\frac{\partial V_{f}}{\partial t}+\rho_{f}\left(V_{f}-W_{f}\right)∙\nabla V_{f}-\nabla \cdot \tau =0, \end{array}$$

where *V*_*f*_  is the velocity vector,  *ρ*_*f*_ is the fluid density, and *W*_*f*_ is the moving coordinate velocity, respectively. *τ* is the fluid stress tensor:

(2) $$\begin{array}{c} \tau =-P\delta_{ij}+2\mu {\dot{\gamma }}_{ij}. \end{array}$$

where *P* is the pressure, *δ*_*ij*_ is the Kronecker delta, *μ* is the viscosity function, and ${\dot{\gamma }}_{ij}$ is the strain rate:

(3) $$\begin{array}{c} {\dot{\gamma }}_{ij}=\frac{1}{2}\left.\left(\nabla V+\nabla V^{T}\right.\right). \end{array}$$

In the present study, we assumed that the shear thinning behavior of blood is described by the Carreau model as follows:

(4) $$\begin{array}{c} \mu =\mu_{\infty }+\left(\mu_{0}-\mu_{\infty }\right){\left(1+A{\dot{\gamma }}^{2}\right)}^{n}, \end{array}$$

where $\dot{\gamma }$ is the shear rate (the second invariant of ${\dot{\gamma }}_{ij}\;$), *μ*_0_ and *μ*_∞_ are the zero and infinite shear rate limit viscosities, respectively, *μ*_0_ = 0.056 Pa.s, and *μ*_∞_ = 0.0035 Pa.s. *A* and *n* are the model constants which are equal to 10.976 and −0.4, respectively [2]. The density of blood was taken to be *ρ*_*f*_ = 1050 *kg*/*m*^3^ [28].

The governing equation for the solid domain can be described by the elastodynamics equation:

(5) $$\begin{array}{c} \rho_{s}{\ddot{d}}_{s}=\nabla_{0}\cdot \sigma_{s}+\rho_{s}f_{s}, \end{array}$$

where *ρ*_*s*_ is the wall density, *σ*_*s*_ is the Cauchy stress tensor, $\ddot{d_{s}}\;$is the local acceleration of the solid, and *f*_s_ is the body force per unit volume, which is equal to zero. Since the mechanical deformations are not large, the arterial wall and fibrous cap were assumed to be linear elastic [29, 30] with density *ρ*_*s*_ equal to 1300*kg*/*m*^3^ [31], a Young's modulus of 1 MPa and Poisson's ratio of 0.49. The lipid pool is much softer than the plaque and it was assumed to be linear elastic with a Young's modulus of 50 kPa [32], Poisson's ratio of 0.499, and density of 920 *kg*/*m*^3^ [33].

### 2.2. Fluid-Structure Interaction

In order to get a fully coupled Fluid-Structure system, fluid and solid domains are coupled in the arterial wall boundary by the following boundary conditions:

(6) $$\begin{array}{c} \text{Displacement}:\;d_{f}=d_{s}s,\\ \begin{array}{c} \text{Traction}:n.\sigma_{f}=n.\sigma_{s,} \end{array}\\ \begin{array}{c} \text{No slip}:d_{f}^{.}=d_{s}^{.} \end{array}, \end{array}$$

where *d*, *σ*, and *n* are the displacement vectors, stress tensor, and normal vector, respectively.

### 2.3. Geometry and Boundary Conditions

Study by Li et al. [34] shows that the stenosis severities between 30% and 70% are most critical regarding the thickness of the fibrous cap. 10% to 30% are not critical at all and 70%–95% are dangerous no matter what is the thickness of the fibrous cap. Thus, we use 30% to 70% severities in the present study.

**Figure 1:**
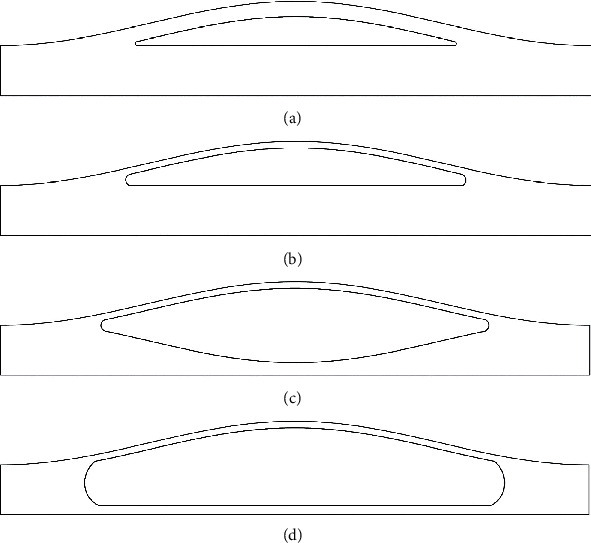
Geometry of a 50% stenosis and the pattern of lipid extension into the arterial wall. (a) G1: thick fibrous cap with smaller lipid core. (b) G2: plaque with thin fibrous cap and no extension of lipid core into the arterial wall. (c) G3: normally extended. (d) G4: overly extended lipid core.

The geometry of the stenosis was defined by Equation (7). A total of 20 models were categorized in four groups based on the volume of their lipid core. Group 1 (G1) and 2 (G2) consist of the stenosis models (30%–70%) without the extension of the lipid core into the arterial wall. The fibrous cap thickness is 150 *μ*m in group 1 and 65 *μ*m in group 2. Group 3 (G3) and 4 (G4) consist of the models with 65 *μ*m fibrous cap thickness and a large lipid core that extended into the arterial wall (Figure 1). Axisymmetric models with severities of 30% to 70% by cross-sectional area were created using the Matlab software (Mathworks, Massachusetts, USA) and ADINA (V 9.0, ADINA R&D Inc., Watertown, MA).

(7) $$\begin{array}{c} r_{\left(z\right)}=-R_{0}\left[1-\left(\frac{\delta }{D}\right)\left(1+\cos\left(\frac{2\pi z}{z_{0}}\right)\right)\right], \end{array}$$

where *D* = 3 mm, *Z*_0_ = 6 mm (2D), and *δ* are the diameter of the lumen in stenosis throat [35].

**Figure 2:**
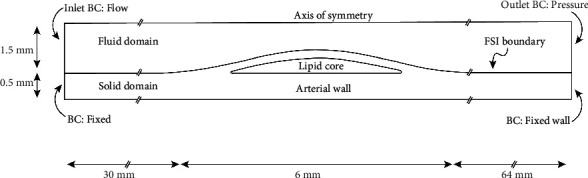
Complete fluid-structure model with the respective boundary conditions and dimensions.

Figure 2 shows the full axisymmetric model defined with the respective boundary conditions and dimensions. The fully developed pulsatile velocity profile of the right coronary artery was used as the inlet boundary condition, and the pressure pulse was applied as the outlet boundary condition. Both the inlet and outlet boundary conditions were used by Sadeghi et al. [35], each boundary condition corresponds with a different range of stenosis severity. The velocity and pressure profiles in the inlet and outlet boundaries, respectively, are reported in Figure S1 in supplementary materials. No slip condition was assumed on FSI boundary. The arterial wall at the inlet and the outlet were fixed, and the prestenotic and poststenotic regions were long enough to avoid boundary conditions effects. The interface of the lipid core-arterial wall was modelled with the continuity condition of displacement and equal stress in the boundary between vessel wall and the lipid core [36].

### 2.4. Numerical Method

A direct two-way coupling method was used for Fluid-Structure Interaction simulations using ADINA 9.0 software. This method combines the matrices of both fluid and solid region during the solution process. Both force and displacement/velocity were used as the coupling convergence criteria. The relative tolerances for both criteria were set to 0.001. Newton-Raphson and Euler method were used for linearization process and time-integration, respectively. A Sparse matrix solver that is originally based on the Gaussian Elimination method was utilized for the solution process.

Three node triangular elements were used to discretize wall, plaque, and lipid pool. For the fluid region 4 node tetrahedral elements were used. The density of the meshes increases as they approach the vessel wall to capture the changes inside the boundary layer. In order to study the mesh independence, multiple simulations with varying number of elements were done for 70% stenosis model in each main group. As seen in Table 1, 11400 elements for the fluid domain and 145393 elements for the solid part are enough to achieve mesh independence for the G4 models.

As it is indicated in Table 1, higher mesh densities than 11400/145393 yields results with less than 1.5% change in velocity in the stenosis neck and circumferential stress in the stenosis apex in respect to the previous mesh density; thus, in order to reduce the computational costs and run time for the simulation, we have used 11400/145393 mesh number.

In order to achieve the fully developed pulsatile flow (Womersley solution), 4 complete cardiac cycles were used. Since the results for the fourth and third cycle were not different, the results from the fourth cycle were used for the analysis.

As previous publication [35], a time step size of 0.005 s was used for the simulations. Also, we used an automatic time stepping (ATS) method that controls the time step size in order to obtain a converged solution [37].

## 3. Results and Discussion

In previous publications, we have performed several checks for the accuracy of the ADINA FSI results and found satisfying agreement between the ADINA results and the experimental and numerical results obtained by other researchers [35].

In this study, to investigate the effect of the extended lipid core on the hemodynamic parameters, we used a total of 20 models that were categorized in 4 groups in respect to their different lipid core size. Group 1 and 2 correspond to the lesions without extended lipid core, whereas in group 3 and 4, lipid core has extended into the arterial wall.

**Figure 3:**
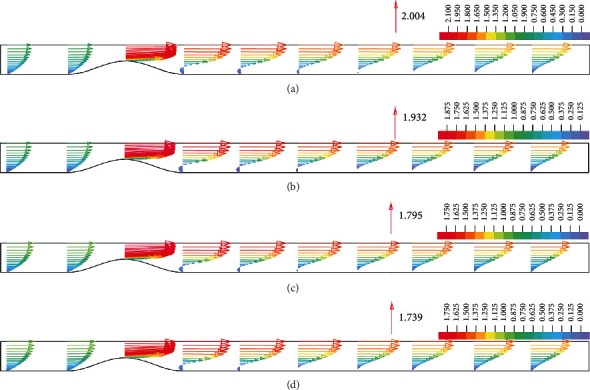
Velocity profiles **)**m/s**(** for the model of 70% stenosis in 10 points starting 1D before the base of the stenosis to 6D after the stenosis: (a) Group 1 .(b) Group 2 .(c) Group 3 .(d) Group 4.

Figure 3 shows the velocity profiles at the peak flow rate for the models of G1, G2, G3, and G4 with 70% stenosis. As seen in Figure 3, the maximum velocity occurs at the stenosis throat and its value decreases with the increase in lipid core size. The maximum velocity values at the vicinity of the stenosis throat at peak flow rate are summarized in Table 2 for all of the models. According to Table 2, the maximum velocity increases as the stenosis become more severe in each group which leads to a decrease in the intraluminal pressure. The values of the intraluminal pressure at the stenosis throat at the peak flow rate are summarized in Table 3. These results show that with the increase of the stenosis severity, the pressure decreases at the stenosis throat. Also, the pressure increases as lipid core size increases and extends into the arterial wall. These change in the pressure field directly influence the wall's and the plaque's mechanical stresses.

**Figure 4:**
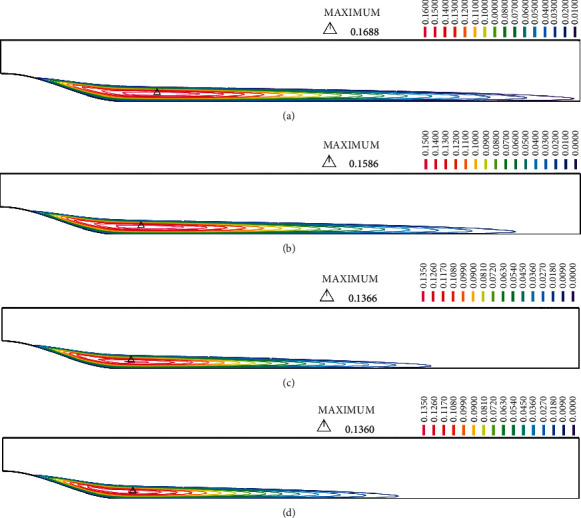
Reversal flow streamlines (m/s) at peak flow rate for 70% stenosis in (a) group 1 (b) group 2 (c) group 3 (d) group 4.

In addition, according to Table 2, the maximum velocity increases as the stenosis become more severe in each group which leads to a decrease in the intraluminal pressure. Maximum velocity in each group increases due to the increase in stenosis severity. The increase in maximum velocity due to increased stenosis severity is explained by the reduced lumen cross sectional area. However, increasing the size of lipid core results in higher wall displacement and increase the lumen cross-sectional area at the stenosis throat (this increase for models with 70% stenosis severity is approximately equal to 11.71% for G1, 16.14% for G2, 25.80% for G3, and 29.99% for G4) which leads to lower maximum velocity. While decreasing the size of lipid core results in lower wall displacement at the stenosis throat which leads to higher maximum velocity especially for higher stenosis severity. Because of the change of the velocity field around the stenosis, the pressure field was also changed. Due to acceleration of flow in stenotic region, pressure drops at the proximal shoulder and throat of stenosis. Distal to stenosis, flow decelerates, and there is a tendency for pressure recovery. The adverse pressure gradient between the stenosis throat and the distal side of the stenosis causes the boundary layer separation and the subsequent formation of a recirculation region in the distal shoulder and poststenotic areas. As seen in Figure 3, the length of this region decreases with increasing lipid core size. To better compare the recirculation regions, Figure 4 shows the reversal flow streamlines for 70% stenosis at peak flow rate time point. As it is shown in Figure 4, an increase in the lipid core size causes the length of the recirculation region to decrease. A study on LDL mass transfer in stenosed arteries has shown that decrease of the recirculation region in the distal shoulder of the stenosis leads to higher LDL accumulation on the arterial wall that can result in higher lipid infiltration into the lesion [38]. Higher LDL infiltration into the plaque may lead to further lipid core expansion.

As it was mentioned before, due to the flow acceleration in the stenosis throat, a decrease in the intraluminal pressure is expected. As it is seen in Table 3, as the stenosis severity increases, the pressure further decreases at the throat of stenosis. Also, an increase in the lipid core size and its further extension to the arterial wall causes pressure to increase. These blood pressure variations directly influence the displacements and stresses in the wall.

**Figure 5:**
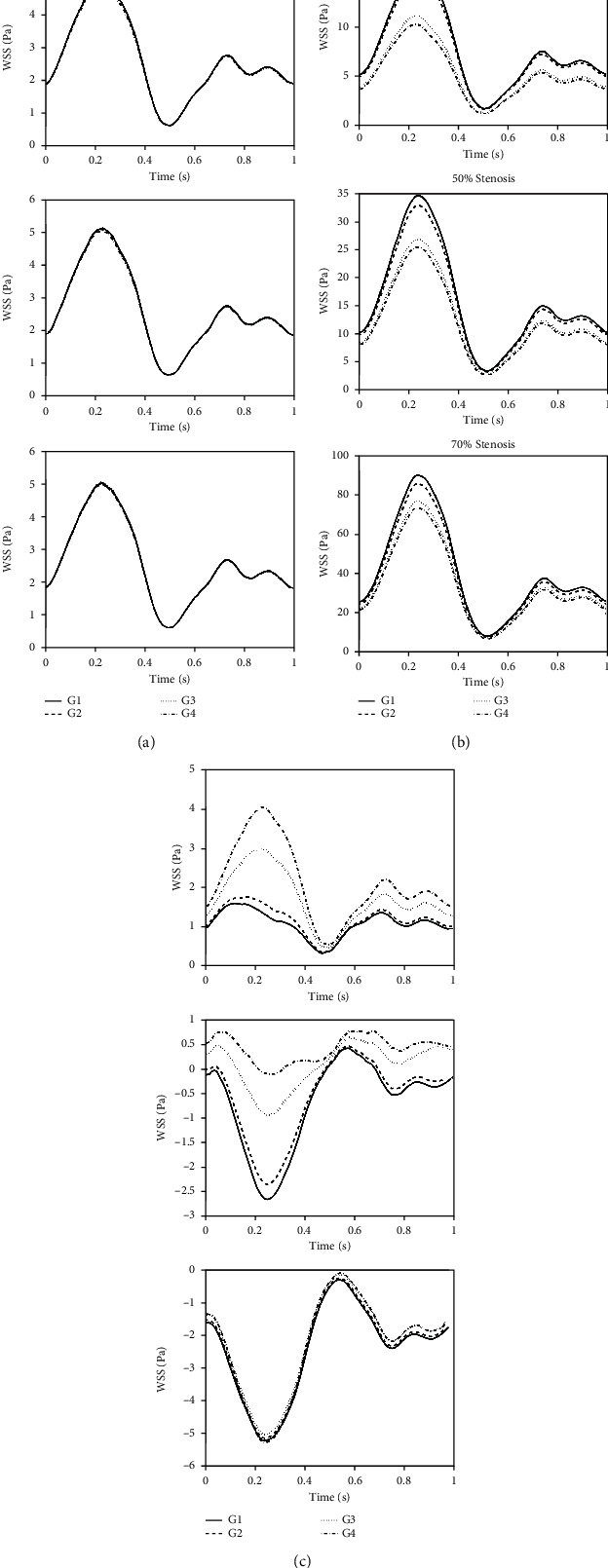
Wall shear stress at (a) proximal, (b) apex, and (c) distal of the stenosis. The beginning and the end points of the stenosis profile were taken as proximal and distal points. Peak of the stenosis profile was selected as the apex.

Figure 5 shows the WSS variations during one pulse at three points at the proximal base, apex, and end of the plaque for models with stenosis severities of 30%, 50%, and 70%. It must be noted that the beginning, the end, and the peak points of the stenosis profile were taken as proximal, distal, and apex points, respectively. Peak of the stenosis profile was selected as the apex. As seen in Figure 5, there is no significant difference in WSS in the proximal point between models from G1 to G4. But in the stenosis apex, when lipid core extends to the arterial wall, not only the mean and maximum values of WSS decrease but also its pulse amplitude decreases. Decrease of the maximum value of the WSS in models of G3 and G4 indicates that the erosion risk is lower in plaques with extended lipid core in the arterial wall (G3 and G4) than that of the models with low lipid content (G1) or without the extended lipid core (G2). Previous studies have shown that the WSS values higher than 40 Pa and 100 Pa are able to damage and strip the ECs, respectively [20, 21].

In addition, our results showed that WSS at the end of the stenosis increased in models with 30% and 50% stenosis due to the increased lipid core size, whereas there was no significant difference in models with 70% stenosis.

**Figure 6:**
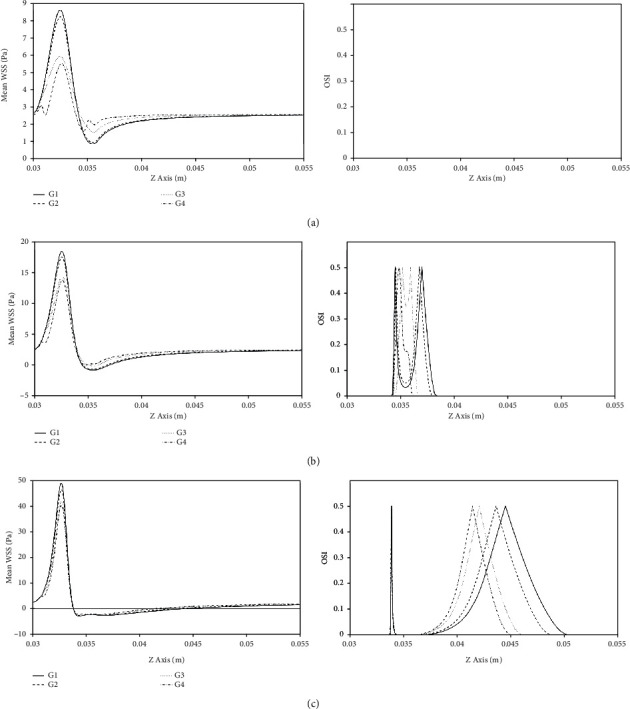
Mean WSS and OSI for (a) 30% stenosis, (b) 50% stenosis, and (c) 70% stenosis.

Figure 6 shows the distributions of time averaged WSS, so called mean WSS, and the oscillatory shear index (OSI) along the arterial wall for the 30%, 50%, and 70% stenosis severities. The mean WSS is defined as:

(8) $$\begin{array}{c} \langle \tau \rangle =\frac{1}{T}\int_{0}^{T}\tau \;dt. \end{array}$$

The OSI is another hemodynamic parameter that was introduced by He and Ku [39] and has been used for identifying the regions with oscillatory shear stress. OSI is calculated using the following equation.

(9) $$\begin{array}{c} OSI=\frac{1}{2}\left(1-\frac{\left|\langle \tau \rangle \right|}{\langle \left|\tau \right|\rangle }\right), \end{array}$$

where |*τ*| is the WSS magnitude and 〈*τ*〉 is the mean WSS.

The range of OSI is 0 ≤ OSI ≤ 0.5, where 0 corresponds to unidirectional flow and 0.5 to purely oscillatory flow. The points with OSI value equal to 0.5 represent the time averaged flow separation and reattachment points.

As seen in Figure 6, the peak mean WSS increases as stenosis severity increases. The high mean WSS happens in the proximal shoulder and apex of plaque. The comparison between models G1, G2, G3, and G4 with stenosis severities of 30%, 50%, and 70% shows that models with smaller lipid core have higher values of mean WSS around the plaque. In vitro studies have shown that platelet activation, adhesion, and aggregation are greatest in areas of high WSS [40]. Moreover, high WSS stimulates nitric oxide (NO) production by ECs, which suppress smooth muscle cell (SMC) proliferation and induce apoptosis of SMCs [41]. Furthermore, due to the inflammatory state of the lesion, NO can stimulate the production of MMPs by macrophages. These changes can lead to thinning of the fibrous cap and thus increase the risk of plaque rupture [42]. At the distal region of the stenosis, as the stenosis severity increases, the mean WSS decreases and the recirculation zone is formed and developed.  Table 4 indicates the time averaged recirculation zone length for all models. It is observed from Table 4 that the time averaged recirculation zone length increases as the stenosis severity increases. Also, an increase in the lipid core size causes the time averaged recirculation zone length to decrease [43]. As seen in Table 4, models of G3 and G4 have smaller mean recirculation zones. Previous studies have shown that the lumen surface concentration (LSC) of LDLs increases as the mean recirculation length decreases [40]. On the other hand, the comparison between models G1, G2, G3, and G4 with 50% stenosis shows that the mean WSS magnitudes for models G3 and G4 are lower than that of models G1 and G2. Low mean WSS increases EC turnover rate and therefore increases the number of leaky junctions [44]. The increased number of leaky junctions in the low WSS area leads to higher infiltration of the atherogenic particles [45]. Previous studies have shown that low WSS increases the expression of intercellular adhesion molecule-1 (ICAM-1), vascular cell adhesion molecule-1 (VCAM-1), and monocyte chemotactic protein-1 (MCP-1) by ECs. While ICAM-1 and VCAM-1 mediate the rolling and adhesion of monocytes on the EC surface, MCP-1 promotes transmigration of monocytes into the intima [8]. Therefore, increased LSC and transmigration of monocytes into the artery wall at the distal shoulder of stenosis in models of G3 and G4 will lead to further plaque growth and lipid core expansion in these models. Continued plaque growth leads to severe stenosis and moves the mean separation point towards the stenosis apex and the mean reattachment point downstream as it can be seen in Figure 5 which is in good agreement with Fazli et al. [38]. The OSI distribution also shows that there is no oscillatory WSS region in models with 30% stenosis. But in models with 50% stenosis, an oscillatory WSS area appears on the distal shoulder and poststenotic area. As seen in Figure 5, the length of this area on the distal shoulder of models G3 and G4 is larger than models G1 and G2. But at the poststenotic area, the oscillatory WSS area is larger in models G1 and G2 than models G3 and G4. Himburg et al. [46] introduced the relative residence time (RRT) index which includes the effects of both mean WSS and OSI and is defined by the following equation:

(10) $$\begin{array}{c} RRT\approx {\left[\left(1-2\times OSI\right)\langle \left|\tau_{w}\right|\rangle \right]}^{-1}. \end{array}$$

Calculating this parameter on the distal shoulder of the plaques with 50% stenosis shows that RRT significantly increases due to the increase in lipid core size. For example, RRT at the point of *Z* = 35 mm for G1, G2, G3, and G4 equals to 1.492, 1.908, 10.821, and 19.898, respectively. In the regions with high RRT, the blood particles have enough time to infiltrate the wall [46]. Consequently, a higher growth rate of plaque and further lipid core expansion is expected in models G3 and G4 compared with G1 and G2. In models with 70% stenosis, the oscillatory WSS region on the distal shoulder decreases and moves towards the stenosis apex, while the other oscillatory WSS region moves downstream as it can be seen in Figure 6. The oscillatory WSS regions in the poststenotic area, which are the possible areas of formation of new lesions, are closest and farthest in G4 and G1, respectively. Therefore, the possible area of formation of new lesion is farther from the stenosis in plaques with smaller lipid core.

**Figure 7:**
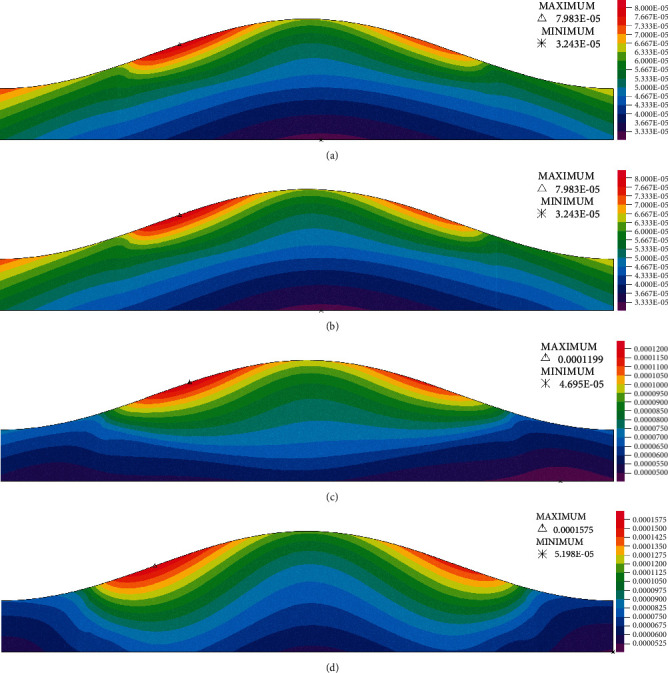
Wall displacement (m) contours for (a) G1, (b) G2, (c) G3, and (d) G4 models with 70% severity.

Wall radial displacement contours are shown in Figure 7 at the peak flow rate (t/*T* = 0.25). Full set of wall radial displacement contours are presented in Figure S2 in supplementary materials. The maximum displacement in models with lower stenosis severities (30%–60%) shifted from the prestenotic region to the stenosis apex due to the increase in lipid core size. However, while the stenosis getting more severe (≥60%) the maximum displacement that is located on the prestenotic region on G1 models shifts to the shoulders as the intraluminal pressure in the stenosis throat decreases. As it was mentioned before the velocity and the intraluminal pressure is also affected by the wall displacement in the stenosis throat. The radial displacement for the stenosis apex has been summarized in Table 5. As seen in Table 5, radial displacement increases in each severity due to the increase in lipid core size [47], making a larger lumen area and thus reduce the maximum velocity at the stenosis throat.

**Figure 8:**
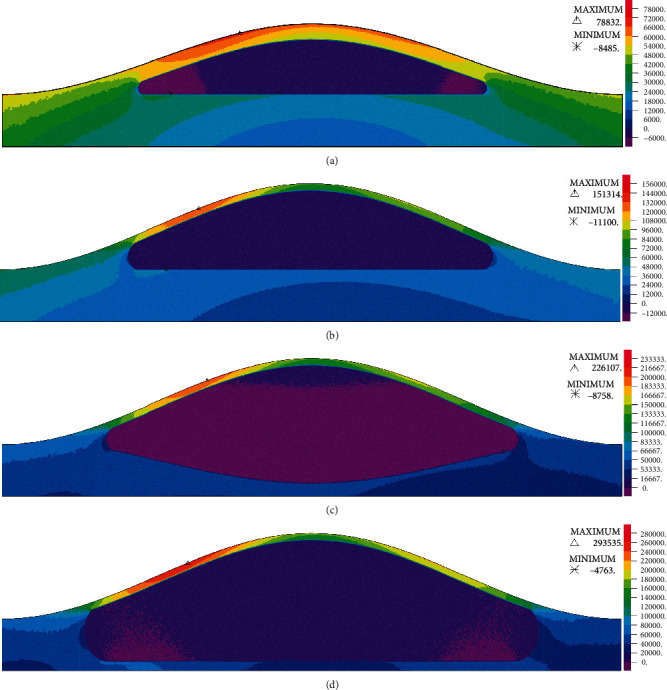
Circumferential wall stress (Pa) for (a) G1 (b) G2 (c) G3 (d) G4 models with 70% stenosis.

It has been shown in the previous studies that high wall mechanical stresses are partly responsible for the plaque rupture [44, 45, 48, 49]. Circumferential wall stress (CWS) is considered as the most important component of the stress tensor in assessment of plaque rupture as it is the most dominant one. CWS contours at the peak flow rate for models of G1 to G4 with 70% stenosis are presented in Figure 8. Full set of CWS contours are presented in Figure S3 in supplementary materials. Maximum CWS values are also summarized in Table 6. Results show that the maximum CWS in lower stenosis severities (30%–40%) is located on the arterial wall in G1 models since the lipid core is not big enough to affect the fibrous cap. As for the G2, and G3 models and in lower stenosis severities of G4, maximum CWS is located on the stenosis apex. As stenosis severity increases (≥70%) in G4, the maximum CWS shifts from stenosis apex to the proximal shoulder. This may be due to the increase in the axial forces that is exerted by the higher pressure gradient forces on the surface of the stenoses with higher severity and the reduction in the intraluminal pressure at the stenosis throat [50, 51]. A recent study by Toutouzas et al. [52] using optical coherence tomography found that 34.5% of the plaque ruptures that have led to MI happened on the stenosis apex while 65.4% was related to the lateral shoulders (34.5% proximal and 30.9% distal). The data presented in Table 6 also indicate that the maximum CWS in G1 and G2 increases due to the increase in severity which is in good agreement with the study of Galaz et al. [29]. Comparing G1 and G2, the maximum CWS increases in respect to the increase in lipid core size and decrease in the fibrous cap thickness (mean CWS of 95956 ± 17.76% in G2 compared with 71291 ± 6.76% in G1). This increase in maximum CWS is also due to the aforementioned increase in intraluminal pressure.

The results for models of G3 show that with the penetration of the lipid core to the arterial wall, maximum CWS increases significantly (approximately 100%) compared with G1 and G2. Note that although the intraluminal pressure is increased in G3 compared with G1 and G2, the drastic increase in the CWS is more related to the increase in lipid core size and its penetration into the arterial wall.

As the lipid core size increases and extends to the arterial wall (G4), the maximum CWS increases significantly compared with G1 and G2 (approximately 200% compared with G2 and 300% with G3). This is due to the thinning (weakening) of the underlying arterial wall. It is also evident that with the penetration of lipid core into the arterial wall, the maximum CWS happens in 40% and 30% severities for G4. This is consistent with the findings of the previous studies showing that thrombotic coronary occlusions mostly happen in plaques with stenosis less severe than 50% [50, 51].

The results show that unlike models in G1 and G2, the maximum CWS decreases due to growing severity in G4. Finet et al. [53] used an image-based finite element method that showed in realistic atherosclerotic plaques with an expanded lipid core that the maximum wall stresses decrease in respect to the stenosis severity that verifies our results. As mentioned before, the intraluminal pressure decreases due to the increase in stenosis severity in all G1, G2, G3, and G4 models. Keeping in mind the intraluminal pressure and maximum CWS values, it is evident that the maximum CWS increases in respect to decrease in intraluminal pressure (at minimum lumen area) in models without the extended lipid core, while it decreases due to the decreasing intraluminal pressure in plaques with an extended lipid core that weakened the underlying arterial wall.

## 4. Conclusions

In this study, in order to understand the effect of the extension of lipid core into the arterial wall on the variation of the hemodynamic parameters, pulsatile blood flow through a stenotic coronary artery was simulated by taking into account the FSI. Stenosis models with severities of 30%–70% stenosis were studied in 4 main groups (G1, G2, G3, and G4) using a finite element method.

Hemodynamic parameters results showed that the extension of lipid core into the arterial wall leads to decreased maximum velocity at the stenosis throat and reduced length of the recirculation region that is formed at the distal shoulder and downstream of the plaque. Additionally, not only it leads to decreased mean and maximum values of WSS but it also leads to decreased pulse amplitude of WSS at the stenosis throat. In moderate stenoses, the extension of lipid core into the arterial wall decreases mean WSS magnitude and increases the length of the region of oscillatory WSS on the distal shoulder. In more severe stenoses, it reduces the length of the region of oscillatory WSS and moves it toward the apex of the plaque. These results indicate that the extension of the lipid core into the arterial wall favors further progression of the plaque and growth of the lipid core. The extension of the lipid core into the arterial wall results in the increased radial displacement at the stenosis apex and increased WCS at the fibrous cap of the proximal shoulder of the plaque.

## Figures and Tables

**Table 1:** Mesh independence study results for 70% stenosis from G4.

Number of elements (fluid/solid)	Velocity in the stenosis inlet (m/s)	Circumferential stress at stenosis apex (pa)
8700/105948	0.98411	242777
9900/134776	0.98400	242030
11400/145393	0.98394	242834
12900/233422	0.98392	239527
14100/323502	0.98396	235658

**Table 2:** Maximum velocity at the stenosis throat at the peak flow rate.

Percentage	G1 (m/s)	G2 (m/s)	G3 (m/s)	G4 (m/s)
30%	1.12	1.10	1.05	1.02
40%	1.21	1.1	1.12	1.08
50%	1.35	1.32	1.23	1.19
60%	1.59	1.54	1.43	1.38
70%	2.00	1.93	1.79	1.73

**Table 3:** Intraluminal pressure at the stenosis throat at the peak flow rate.

Percentage	G1 (kPa)	G2 (kPa)	G3 (kPa)	G4 (kPa)
30%	15.05	15.06	15.11	15.13
40%	15.01	15.02	15.09	15.12
50%	14.88	14.91	14.99	15.03
60%	14.69	14.73	14.83	14.88
70%	14.42	14.50	14.62	14.68

**Table 4:** Mean length of reversal flow region.

Percentage	G1 (mm)	G2 (mm)	G3 (mm)	G4 (mm)
30%	0	0	0	0
40%	0	0	0	0
50%	2.49	2.19	0.74	0
60%	5.28	4.79	3.60	3.21
70%	10.71	9.83	8.25	8.13

**Table 5:** The radial displacement in the stenosis throat at peak flow rate.

Percentage	G1 (mm)	G2 (mm)	G3 (mm)	G4 (mm)
30%	0.059	0.073	0.158	0.198
40%	0.059	0.074	0.149	0.184
50%	0.057	0.074	0.137	0.165
60%	0.053	0.071	0.120	0.142
70%	0.046	0.063	0.099	0.115

**Table 6:** Maximum circumferential wall stress, mean value and coefficient of variance at peak flow rate.

Percentage	G1 (pa)	G2 (pa)	G3 (pa)	G4 (pa)
30%	67888	75828	206109	306921
40%	67928	84510	213929	305417
50%	68371	95322	211447	294335
60%	73438	104821	208493	280344
70%	78832	119298	207702	279753
Mean (pa) + CV (%)	71291 + 6.76%	95956 + 17.75%	209536 + 1.49%	293354 + 4.46%

## Data Availability

All data used to support the findings of this study are included within the article.

## Nomenclature

d: Displacement vector
P: Pressure
*δ*: Diameter of the lumen in stenosis throat
$\ddot{{\text{d}}_{\text{s}}}$: Local acceleration of the solid
n: Normal vector
V_f_: Velocity vector
W_f_: Moving coordinate velocity
f_s_: Body force per unit volume
*ρ*_*f*_: Fluid density
*δ*_*ij*_: Kronecker delta
*μ*_0_: Zero shear rate limit viscosities
*μ*_∞_: Infinite shear rate limit viscosities
$\mu \dot{\left(\gamma \right)}$: Viscosity function
${\dot{\gamma }}_{ij}$: Shear rate (the second invariant of deformation tensor)
*ρ*_*s*_: Wall density
*σ*_*s*_: Cauchy stress tensor
*σ*: Stress tensor
|*τ*|: WSS magnitude
〈*τ*〉: Mean WSS
*τ*: Fluid stress tensor.

## Supplementary Materials

Supplementary Materials for a complete image results of the boundary conditions and wall radial displacement and CWS please refer to Figures S1, S2, and S3.
