# Supplementary material for: Effect of Extended Lipid Core on the Hemodynamic Parameters: A Fluid-Structure Interaction Approach
Source: Appl Bionics Biomech. 2022 Mar 17;2022:2047549. doi: 10.1155/2022/2047549 (PMC8947935; doi:10.1155/2022/2047549)
Supplement: Supplementary Materials — for a complete image results of the boundary conditions and wall radial displacement and CWS please refer to Figures S1, S2, and S3. [file 2047549.f1.docx]

| (A) | (B) |
| --- | --- |
|  |  |
| Figure S1. Boundary conditions for different stenosis severities (A) Inlet velocity (m/s) (B) Outlet pressure | |





Figure S2. Wall displacement contours for G1 (A), G2 (B), G3 (C), G4 (D) with different stenosis severities.

a



Figure S3. Wall circumferential stress contours for G1 (A), G2 (B), G3 (C), G4 (D) with different stenosis severities.
